# Supplementary material for: Disaster and distress: The double burden. Depression, anxiety, and post-traumatic stress disorder in doctors during the COVID-19 pandemic in Pakistan
Source: PLOS Ment Health. 2026 Jan 29;3(1):e0000421. doi: 10.1371/journal.pmen.0000421 (PMC12854420; doi:10.1371/journal.pmen.0000421)
Supplement: Attachment — Submitted filename: Response to Reviewers.odt [file pmen.0000421.s002.odt]

PMEN-D-25-00130
Disaster and Distress: The Double Burden.

Depression, Anxiety, and Post-Traumatic Stress Disorder in Doctors during the COVID-19 Pandemic in Pakistan
PLOS Mental Health

Dear Editor and Reviewer #3, 

We are sincerely thankful to the reviewer for taking out time to review our manuscript and provide valuable suggestions. We carefully considered the feedback and suggestions and have revised the manuscript accordingly. We have provided a detailed, point-by-point response to the reviewer suggestions as below:

The manuscript addresses a relevant and timely topic, exploring mental health among physicians during the COVID-19 pandemic in a lower-middle-income country. Although it employs standardized instruments (PHQ-9, GAD-7, IES-R) previously validated in Pakistan, the limitations section fails to discuss potential bias arising from the use of English versions of these tools, despite the availability of validated Urdu versions.
The conclusion would be stronger and more persuasive if multivariable models were applied to control for potential confounding factors. This analytical limitation should be explicitly acknowledged and addressed in the revision.

We recognize that using the English versions of the PHQ-9, GAD-7, and IES-R may introduce some language-related bias to our study. While doctors in Pakistan are generally fluent in English and the English versions of the stated research scales or instruments are widely used in Pakistan, the validated Urdu translations might have provided better cultural alignment. We have also acknowledged in the manuscript that we did not use multivariable regression to adjust for confounding. We have addressed the fact that our non-normally distributed data would only meet the assumptions of logistic regression modelling, and because our outcomes had ordered severity levels, running logistic models would have required collapsing (such as to two categories of each outcome in case of binary logistic regression) or reshaping these categories and could have reduced important clinical details, thereby potentially distorting the results (Line numbers 374-382). 

We thank the reviewer again for the feedback and suggestions. 

Kind Regards, 

Madah Fatima, 
Corresponding Author.
