# Supplementary material for: Disaster and distress: The double burden. Depression, anxiety, and post-traumatic stress disorder in doctors during the COVID-19 pandemic in Pakistan
Source: PLOS Ment Health. 2026 Jan 29;3(1):e0000421. doi: 10.1371/journal.pmen.0000421 (PMC12854420; doi:10.1371/journal.pmen.0000421)
Supplement: Attachment — Submitted filename: R2 Response to Reviewers.odt [file pmen.0000421.s004.odt]

Dated 10-01-2026
PMEN-D-25-00130R2
Disaster and Distress: The Double Burden.

Depression, Anxiety, and Post-Traumatic Stress Disorder in Doctors during the COVID-19 Pandemic in Pakistan
PLOS Mental Health

Dear Editor and Reviewer, 

We are sincerely thankful to the reviewer for taking out time to review our manuscript and provide valuable suggestions and feedback. We carefully considered the feedback and suggestions and have revised the manuscript accordingly. We have provided a detailed, point-by-point response to the reviewer's suggestions as below:

Regarding the response to the methodological concerns, their justification is contextually reasonable, and the transparent discussion of this limitation in the manuscript is acceptable. Concerning the absence of multivariable analyses, it is a methodological standpoint and the limitation is now explicitly framed in the manuscript. 
We thank the reviewer for providing positive feedback to the changes we made by explicitly framing the limitation and transparently discussing it in the manuscript. 
With respect to the conclusion, it is broadly consistent with the study findings; however, some statements would benefit from being slightly tempered to better reflect the cross-sectional design, limited generalizability, and lack of multivariable adjustment. In particular, references to long-term or enduring impact and broad policy implications should be clearly framed as potential or speculative rather than directly inferred from the data.
We are grateful to the reviewer for carefully reviewing the conclusions and providing valuable feedback. This was very helpful, and we realized that some statements needed modification to avoid the extrapolation to policy context, in conclusion, being misconstrued as direct interpretations of the study findings. We have highlighted the limitations in the conclusions section as well, and particularly addressed that broad policy implications and long-term psychological impact are to be interpreted as speculative and not prescriptive or definitive implications of study findings in light of the limitations. (line number 408-429)
We thank the reviewer again for the feedback and suggestions. 

Kind Regards, 
Madah Fatima, 
Corresponding Author.
